# Fully Flexible Textile Antenna-Backed Sensor Node for Body-Worn UWB Localization

**DOI:** 10.3390/s21051641

**Published:** 2021-02-26

**Authors:** Dries Van Baelen, Nicola Macoir, Quinten Van den Brande, Eli De Poorter, Sam Lemey, Jo Verhaevert, Hendrik Rogier

**Affiliations:** Department of Information Technology, Ghent University-imec, Technologiepark-Zwijnaarde 126, 9052 Gent, Belgium; Dries.VanBaelen@UGent.be (D.V.B.); Nicola.Macoir@UGent.be (N.M.); Quinten.VandenBrande@UGent.be (Q.V.d.B.); Eli.DePoorter@UGent.be (E.D.P.); Sam.Lemey@UGent.be (S.L.); Hendrik.Rogier@UGent.be (H.R.)

**Keywords:** wearable sensor systems, flexible electronics, ultra-wideband, UWB, textile antenna, localization, wireless body area network

## Abstract

A mechanically flexible textile antenna-backed sensor node is designed and manufactured, providing accurate personal localization functionality by application of Decawave’s DW1000 Impulse Radio Ultra-Wideband (IR-UWB) Integrated Circuit (IC). All components are mounted on a flexible polyimide foil, which is integrated on the backplane of a wearable cavity-backed slot antenna designed for IR-UWB localization in Channels 2 and 3 of the IEEE 802.15.4-2011 standard (3744 MHz–4742.4 MHz). The textile antenna’s radiation pattern is optimized to mitigate body effects and to minimize absorption by body tissues. Furthermore, its time-domain characteristics are measured to be adequate for localization. By combining the antenna and the bendable Printed Circuit Board (PCB), a mechanically supple sensor system is realized, for which the performance is validated by examining it as a node used in a complete localization system. This shows that six nodes around the body must be deployed to provide system coverage in all directions around the wearer. Even without using sleep mode functionalities, the measurements indicate that the system’s autonomy is 13.3 h on a 5 V 200 mAh battery. Hence, this system acts as a proof of concept for the joining of localization electronics and other sensors with a full-textile antenna into a mechanically flexible sensor system.

## 1. Introduction

Wireless Body Area Network (WBAN) systems are indispensable for the integration of a plethora of useful body-centric functionalities into the Internet of Things (IoT). Currently, they have given rise to healthcare systems providing measurement of life signs, such as blood glucose levels or electrocardiograms (ECGs) [1,2,3]; systems that provide fall detection in the elderly [4,5]; and environmental sensing systems measuring the temperature, gas and dust concentrations, and relative humidity of the surrounding environment for, for example, emergency responders and mining personnel [6]. Among the myriads of possible functionalities, localization is of particular interest to WBAN systems.

Over the years, a multitude of positioning methodologies has been developed, each with their own benefits and disadvantages [7,8]. Among those, the ultra-wideband (UWB) technology described in the IEEE 802.15.4-2011 standard [9] is the most interesting candidate. Here, a very-low-power broadband pulse is applied to provide communication between the tag and anchor. Due to the nature of its signal type and the very wide bandwidth used, the system exhibits high immunity to narrowband signal interference [8]. Furthermore, owing to the very low power present in the signal, which is spread over a large bandwidth, and given the signal’s pseudo-random nature, UWB causes little interference with other wireless technologies [10,11,12]. The high data rate and short packet time cause few opportunities for collisions, allowing for a high node density of the applied system [10,13]. Since the use of large bandwidths causes a very steep flank in the time domain, localization devices can provide very accurate time-of-arrival estimations, down to 10 cm [14]. Finally, because of the very short time-domain pulses, UWB has a high immunity against multipath effects [15,16]. These properties promote UWB technology as a prime candidate for both indoor and outdoor real-time localization systems.

Applications of UWB technology include person proximity detection [17], tracking of cattle [18,19,20] and industrial assets [21], sports analysis [22,23], and many others. Although all these positioning systems use UWB technology, they are still rigid and bulky and, just as is often observed in UWB antenna development, they typically do not take human body proximity into account. To attain true market and customer acceptation, wearable WBAN sensor systems need to be comfortable, discrete, and unobtrusive. This means that they should be low profile and mechanically flexible and that they should spend energy in a responsible, efficient way, since (flexible) battery capacity is a limiting factor in a body-worn system, in particular, when the aforementioned wearability constraints need to remain satisfied.

In this paper, UWB technology is applied to realize accurate localization functionality as a part of a complete mechanically flexible WBAN sensor system, featuring Decawave’s DW1000 localization IC [14]. The system provides sensor data for temperature, relative humidity, and air pressure, which, together with the ranging data, can be stored on-board in a discrete flash memory. The system is implemented on a polyimide foil to provide maximal mechanical flexibility. The bottom of this polyimide foil is then connected to the backplane of a cavity-backed slot antenna, which serves as an integration platform for the discrete electronic components.

The fabrication process in [24] was applied to create a mechanically flexible, full textile cavity-backed slot antenna. Since such low-profile antennas are notoriously narrowband, the broadbanding technique described in [25] was applied. The resulting antenna exhibits a reflection coefficient with respect to 50 Ω lower than −10 dB over both Channels 2 and 3 of the IEEE 802.15.4-2011 standard (3744 MHz–4243.2 MHz and 4243.2 MHz–4742.4 MHz, respectively). Furthermore, the results from [26] indicate that the selected antenna topology is a suitable choice to reduce the phase center shift as a function of incident angle as much as possible, as is required for accurate localization applications. Moreover, [26] proves the feasibility of this antenna topology for on-body deployment, as both its reflection coefficient with respect to 50 Ω and its radiation pattern remain stable under diverse deployment circumstances, such as in free space with and without mechanical bending or when worn on the torso or on the upper right arm of a test person.

To achieve accurate localization performance, both the pulse distortion and the phase center shift of the antenna should be minimized. The former impedes accurate reception of the signal, whereas the latter causes a distance estimation error (DEE). Both parameters are functions of the incident angle of the wave and, hence, must be investigated for all directions relevant for body-worn scenarios. For this, only angles up to $60^{\circ }$ from the antenna’s broadside are considered due to body shadowing effects in directions approaching or exceeding $90^{\circ }$ away from the antenna’s broadside.

The main innovations presented in this paper are as follows:

- Co-design of a mechanically flexible textile antenna for UWB localization and a mechanically flexible PCB featuring both digital and time-domain-sensitive analog parts, and combination of the textile antenna and the PCB into a functional system.
- Proof of the suitability of the full-textile cavity-backed slot antenna for localization purposes by characterizing both the antenna’s pulse distortion and phase center shift.
- Proof of concept of the integration of a textilized unobtrusive body-worn sensor node in an existing UWB localization infrastructure under the presence of body shadowing and proximity.

This paper is structured as follows. Section 2 lists the requirements of the sensor system and the antenna. It specifically highlights the consequences of wearability and of requirements pertinent to localization on a system and antenna design. Furthermore, some feasible localization methodologies are discussed. Section 3 elaborates on various investigated figures of merit. The time-domain behavior of the standalone antenna is discussed, along with the free space power pattern and power consumption of the complete sensor system. Furthermore, the UWB connectivity between the body-worn sensor system and existing localization infrastructure are evaluated. Conclusions are provided in Section 4.

## 2. System Requirements and Design

This section discusses the requirements of the system and elaborates on the design choices made to meet them. The implications for both the antenna and the backbone electronics are investigated. Furthermore, a suitable localization methodology is selected.

### 2.1. System Requirements

WBAN systems should possess a high degree of wearability. This implies that the system should be unobtrusive to the user: it should be lightweight, should have a small footprint, and should be mechanically flexible to interfere as little as possible with the user’s movements. This requirement is also translated into the power source, so frugal use of the available power is essential.

From an electromagnetic point of view, the system should comply with all requirements for efficient and accurate localization in accordance with the IEEE 802.15.4-2011 standard. For this, its antenna is selected to operate in Channels 2 and 3 (3744 MHz–4243.2 MHz and 4243.2 MHz–4742.4 MHz, respectively) of the aforementioned standard. Over both channels, the antenna’s reflection coefficient with respect to 50 Ω should remain below −10 dB while a radiation efficiency of at least 70% should be maintained. Both the radiation pattern and the impedance matching should remain stable over the entire targeted frequency band, and they should not deteriorate when the antenna is deployed on the body. This implies that coupling with the human body should be minimized as much as possible. Not only will this low coupling result in more stable antenna characteristics, remaining comparable to the antenna’s standalone behavior in free space, but also it minimizes the energy absorbed by the wearer’s body. In any practical scenario, external nodes should be able to connect and communicate with the system at all times and for all orientations of the wearer’s body with respect to the surrounding localization infrastructure.

Moreover, the angle-dependent phase difference between a wave received along broadside and a wave incoming from another incident angle introduces a distance estimation error (DEE) when performing flank detection of an incoming pulse. A different way to state this is that the antenna’s phase center shifts as a function of incident angle. For the antenna to be suitable for use in UWB localization systems, it is imperative that the DEE is kept sufficiently small. In other words, the phase center shift as a function of incident angle should remain small. The DEE should remain below 10 cm for all directions relevant to a body-worn scenario defined in Section 1, as this keeps the ranging error introduced by the antenna in the same range as the adopted localization Integrated Circuit’s (IC’s) positioning accuracy.

### 2.2. System Overview

**Figure 1:**
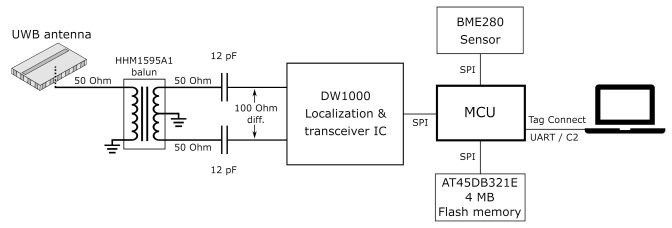
System architecture of the sensor system. The radio-frequency (RF) path of the signal from the antenna towards the localization Integrated Circuit (IC) is elaborated in detail.

Figure 1 outlines the system architecture of the proposed sensor system. The system’s assembly of sensor functionalities may be refitted or expanded at will. To demonstrate this, a basic sensor such as the Bosch Sensortec BME280 temperature, air pressure, and relative humidity sensor [27] is integrated on the board, together with Decawave’s DW1000 localization IC [14], which also hosts a built-in transceiver. Furthermore, the DW1000 module is fully compliant with the IEEE 802.15.4-2011 standard and it can be configured to operate in multiple UWB channels specified in this standard. Both the BME280 sensor and the DW1000 IC are controlled by an ultra-low-power Silicon Labs C8051F921 microcontroller [28].

An Adesto AT45DB321E 4 MB flash memory [29] is included, allowing application-dependent storage of, for example, sensor data. All communication between the aforementioned components occurs via the Serial Peripheral Interface (SPI) protocol.

Since the use of bulky connectors conflicts with the wearability constraints, a landing pad for a Tag Connect [30] interface was provided. The Tag Connect plug features spring-loaded pins to ensure reliable electric contact with the landing pads present on the target PCB. In this way, both the C2 programming interface and the UART bus for runtime communication with a PC are provided.

Finally, the signal from the DW1000 is injected into a cavity-backed slot antenna by a probe feed, as further elaborated in Section 2.3.

### 2.3. Antenna Design

The results from [24,25,26] prove that the cavity-backed slot antenna topology is suitable for the goals stated in Section 2.1. The antenna topology allows for low pulse distortion, and its distance estimation errors induced by a phase-center shift are in the order of 5 cm in all directions when considering a body-worn scenario [26], causing it to qualify for the 10 cm criterion. Furthermore, the frequency-domain characteristics of the topology remain stable when such an antenna is subject to bending or when placed in proximity of a human body [24].

Since the system is designed for smooth integration into clothing, it is desirable for the complete system to be mechanically flexible to hamper the wearer’s motions as little as possible. Therefore, the fabrication procedure and the materials described in [24] are used to create a mechanically flexible, full-textile cavity-backed slot antenna in substrate integrated waveguide (SIW) technology.

**Figure 2:**
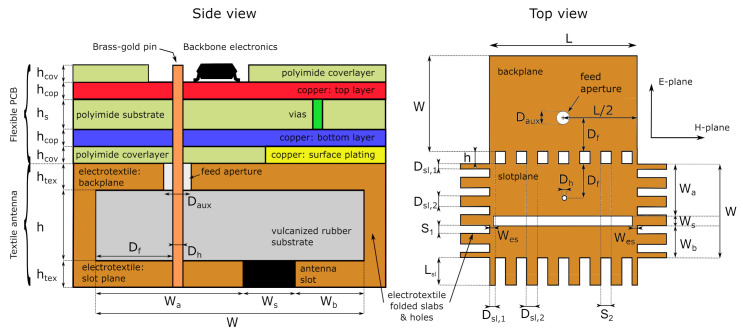
Stackup of the finished sensor node (side view, left) and shape of the unassembled electrotextile patch (top view, right). Dimensions: L = 56 mm, W = 34 mm, W${}_{a}$ = 20 mm, W${}_{b}$ = 10.5 mm, W${}_{s}$ = 4.5 mm, D${}_{f}$ = 15 mm, D${}_{aux}$ = 5 mm, D${}_{h}$ = 1 mm, D${}_{sl,1}$ = 2 mm, D${}_{sl,2}$ = 4 mm, S${}_{1}$ = 2.8 mm, S${}_{2}$ = 4 mm, L${}_{sl}$ = 10 mm, W${}_{es}$ = 1 mm, h = 4 mm, h${}_{tex}$ = 0.15 mm, h${}_{cov}$ = 25 μm, h${}_{cop}$ = 16 μm, and h${}_{s}$ = 50 μm. This model is based on the design from [26], accommodating a different feed pin size.

The antenna is based on the coupled half-mode cavity-backed slot antenna design in [26]. In this topology, the open ends of two differently dimensioned SIW half-mode cavities are placed in close proximity. Together with a sufficiently small separation between the half-mode cavities’ natural resonance frequencies, this close proximity causes both cavities to experience a strong mutual coupling, which triggers mode splitting, as explained in [31]. Through thorough computer-aided design, a suitable choice for the half-mode cavity dimensions and their mutual spacing is found, causing the coupled half-mode cavities to cover the entire desired frequency range. A minor redesign is performed on the antenna from [26], since the integration of the electronics required a comparatively smaller pin diameter. The resulting antenna dimensions are specified in the caption of Figure 2.

The results from [24,25,26] show that the antenna topology exhibits a directive radiation pattern oriented away from the human body, with a measured front-to-back ratio (FTBR) higher than 11 dB within the IEEE 802.15.4-2011 Channels 2 and 3. These results also demonstrate that this antenna topology exhibits low coupling with the human body, thereby reducing the wearer’s Specific Absorption Rate (SAR) and limiting the influence of human body proximity on the radiation pattern and impedance matching of the antenna.

### 2.4. Circuit Design

To provide mechanical flexibility, the electronics forming the backbone functionality of the system are implemented on a flexible polyimide foil, as shown in the side view on Figure 2. The upper part of this schematic concerns the flexible PCB. The lower part of this schematic consists of the textile antenna, bounded by its electrotextile (orange) backplane and slot plane (orange and black). These are surrounded by vertical electrotextile slabs, thus forming the substrate integrated waveguide cavity described in [24] and visualized in the top of Figure 1.

The connection between the antenna and the DW1000 IC is schematically represented in Figure 1, according to the design rules specified in [13]. The RF output of the DW1000 IC is connected to two 12 pF capacitors by means of a 100 Ω differential transmission line. Then, a 50 Ω single-ended transmission line is used to connect each capacitor to a pin of the balanced port of a TDK HHM1595A1 balun [32]. Another 50 Ω single-ended transmission line then connects the balun’s unbalanced port to the antenna feed pin, which is soldered to the transmission line. For this, a trimmed Multi-Contact 42.0001 brass-gold pin with a diameter of 1 mm was selected. The antenna and the circuit’s feed pin structure are co-optimized to provide a 1.8-mm diameter ground clearance, together with an aperture in the backplane of the antenna cavity. Then, the probe feed structure was realized by fitting the feed pin through the middle of this hole, after which the antenna was completed by soldering the pin’s other end to the antenna’s top plane.

**Figure 3:**
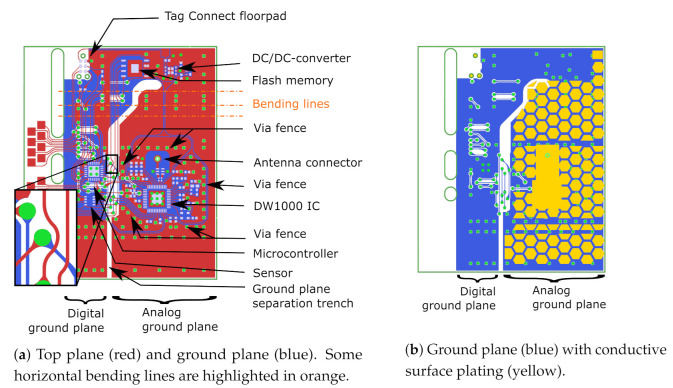
Layout of the Printed Circuit Board (PCB), top view.

When designing mechanically flexible PCBs, care must be taken to distribute the mechanical stress applied to the copper and solder seams as a result of bending. Given that isolated vias and soldering joints are common failure points on flexible substrates, we combined their individual stiffness by aligning vias and component edges to form rigid lines. If sufficient well-positioned bending spaces are implemented in between the rigid lines, the PCB concentrates its bending along those directions in which it is allowed to do so. The result of this design rule is illustrated in Figure 3a, where component edges and vias are aligned as much as possible, while the large footprint provided to the PCB by the back side of the textile antenna provides sufficient spacing to layout the components such that bending lines are realized along which no rigid elements are present. For example, the via rows in between the flash memory and the DW1000 IC are clearly aligned, and sufficient spacing is available in between these components, providing multiple lines over which the PCB can bend. Some examples of these bending lines are highlighted by the orange dash-dotted lines in Figure 3a. Furthermore, bending of the PCB induces mechanical stress that concentrates on the inner corners of the copper planes and on the transitions between signal lines and vias. The ensuing metal fatigue causes a significant risk for the copper to tear at these weak points. This effect is particularly detrimental to the performance of the system, since the cracks may cause signal lines to snap altogether. Therefore, gradual bends in copper sheets are preferred over abrupt inner corners, and the transition between signal lines and vias should be realized in a gradual droplet-like shape. For the same reason, signal lines need to bend smoothly instead of the sharp $90^{\circ }$ or $45^{\circ }$ corners commonly encountered in a conventional PCB design [33]. A correct example is shown in the inset of Figure 3a. Here, the top plane (red) containing all electronics is shown, together with the via layout (green) and the PCB’s ground plane, displayed in blue. Note the gradual bends of the signal lines and the gradual tapering of signal lines approaching a via. In addition, the inner corners of copper surfaces are smoothed to gradual bends.

**Figure 4:**
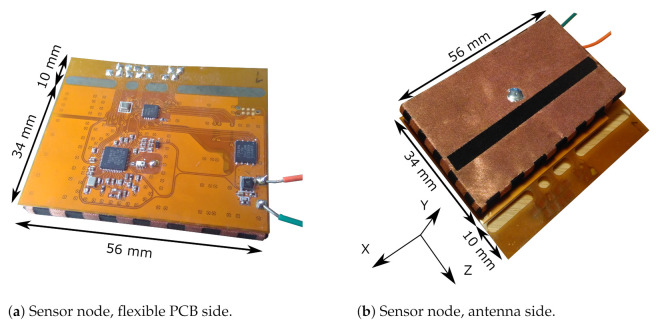
Assembled sensor node. The flexible PCB is attached to the back of the antenna. The H-plane corresponds with the XY-plane, and the E-plane is oriented along the YZ-plane. The size of the antenna is 56 mm × 34 mm. An appendage of width 10 mm has been provided for testing purposes.

The connection between the ground plane and the back side of the antenna is realized by electroless nickel plated gold (ENIG) surface plating (yellow), which is patterned in a honeycomb structure to maintain mechanical flexibility while keeping sufficient electrical contact with the electrotextile beneath it, as is illustrated in the side view on Figure 2. Figure 3b reveals that the left side of the ground plane lacks surface plating. Instead, the 25 μm polyimide coverlayer and the trench visible in the middle of the figure isolate the analog circuitry’s ground plane from noise originating from the digital electronics such as the microcontroller, the sensor, or the flash memory. In addition, these digital components also require protection from the sharp pulses emitted by the DW1000 IC, justifying thorough electromagnetic compatibility (EMC) measures taken concerning and surrounding the DW1000 IC. A via fence was implemented around the DW1000 and its peripheral components, such as the IC’s crystal, decoupling capacitors, and radio-frequency (RF) connection to the antenna to provide additional EMC safety. Over the entire PCB, numerous vias were applied to implement a proper RF system reference plane. The resulting assembled sensor system is shown in Figure 4.

### 2.5. Localization Methodologies

As of today, a multitude of localization algorithms exist. To localize mobile tags, the Two-Way Ranging (TWR) approach is the preferred choice. Here, the mobile tag initiates communication by sending the first message to the anchor, which replies with a message at a set amount of time after reception of the first packet. Based on the time between the transmission of the first packet and reception of the second packet (the so-called round trip delay), the distance between the tag and the anchor node can be calculated. Repeating this process with different anchor nodes provides multiple distance measurements, which in turn can be used to localize the mobile tag. In contrast to, for example, the Time-Difference of Arrival (TDoA) or Time of Arrival (ToA) [8], TWR does not require accurate synchronization between the sensor node and the other localization infrastructure to produce a suitable localization result. By measuring the round-trip delay, TWR avoids this issue, which would otherwise be highly impractical for body-worn localization. TWR has as a further advantage that two nodes can range with each other (proximity detection) without using any external infrastructure. Furthermore, TWR schemes such as Symmetric Double-Sided TWR (SDS-TWR) exhibit excellent resistance against clock-induced ranging errors [13,34]. A disadvantage of TWR in comparison to TDoA- or ToA-based methods is that more messages are required and more complex hardware are needed at the target node. However, this is not prohibitive, and since the need for synchronization between tag and anchor nodes is nonexistent, multi-antenna TWR-based schemes are the preferred choice for body-worn UWB localization.

TWR schemes are perfectly compatible with the IEEE802.15.4-2011 standard used by the DW1000 IC. The standard is an attractive choice for wireless sensor networks (WSNs), since these networks are generally deployed with a large number of nodes (hundreds or even thousands). In addition, having low-cost nodes with long battery lifetime is even more attractive in body-worn systems than it already was for WSNs in general.

## 3. Results

This section discusses the experiments that validate the performance of both the antenna and the complete sensor system with the flexible PCB integrated on the antenna’s backplane. Section 3.1 elaborates on measurements relevant to localization performance that can only be acquired by investigating the antenna itself, such as system fidelity factor (SFF) and distance estimation error. They are therefore performed in free space. Other system parameters can be obtained from the complete sensor system and are presented in Section 3.2. Here, the influence of the presence of a PCB is illustrated by measurement of the power pattern of the PCB, which employs the textile antenna to transmit in continuous wave mode. The system’s performance in body-worn scenarios was then validated by deploying the wireless sensor node in a body-worn positioning experiment. Finally, the system’s power use and the consequences for body-worn design were investigated.

### 3.1. Standalone Antenna

**Figure 5:**
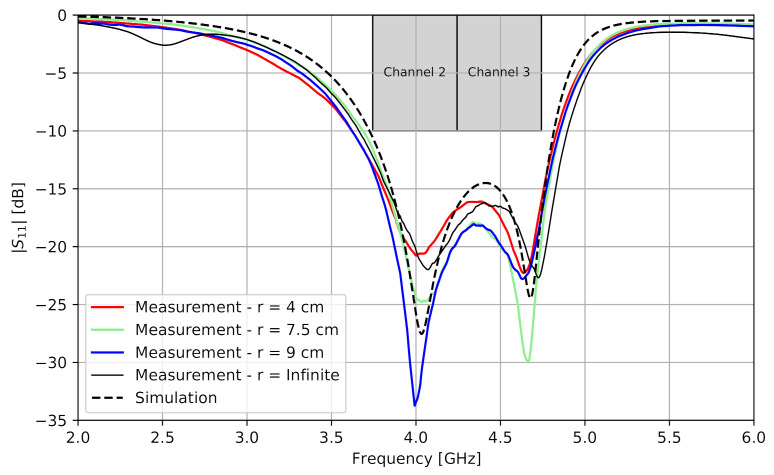
Magnitude of the simulated and measured reflection coefficient $S_{11}$ of the cavity-backed slot antenna as a function of the frequency while subject to mechanical bending along the x-axis in the H-plane and for different bending radii *r*, frequently encountered on the human body.

A prototype of the cavity-backed slot antenna was realized, containing an SubMiniature version A (SMA) connector through which its reflection coefficient $S_{11}$ was measured. Figure 5 compares the magnitude of the free-space-measured reflection coefficient with the simulated one as a function of the frequency. The antenna was also measured under mechanical bending along the x-axis in the H-plane and with bending radii *r*, frequently encountered on the human body. It can be concluded that the antenna is mechanically flexible because the antenna characteristics under bending are similar to the case without bending (indicated as *r* = infinite). In both the measurements and the simulation, Channels 2 and 3 of the IEEE 802.15.4-2011 were also entirely covered.

Electromagnetic field-based UWB link simulations from [26] showed that a system in which this antenna is used both as transmitter and receiver provides a system fidelity factor larger than or equal to 90% for all directions within $60^{\circ }$ of the antenna’s broadside. This means that the pulse distortion introduced by this system remains sufficiently low to allow the localization IC to accurately timestamp the incoming pulse. Furthermore, simulations from [26] predicted that, for these directions, the DEE as a function of incident angle remains in the order of 5 cm. Novel free-space measurements on this antenna confirm that these criteria were met. To prove this, the Orbit/FR DBDR antenna positioning system (MVG, 75005 Paris, France, http://www.orbitfr.com/, accessed on 12 November 2020) and an Agilent N5242A PNA-X Microwave Network Analyzer (Agilent Technologies, Santa Clara, CA 95051, USA, https://www.agilent.com/, accessed on 12 November 2020) [35] were used to obtain the transfer function of the measurement setup, where one port was set at the input connector of the transmit antenna under test and the other port was positioned at the output connector of an identical receive antenna.

**Figure 6:**
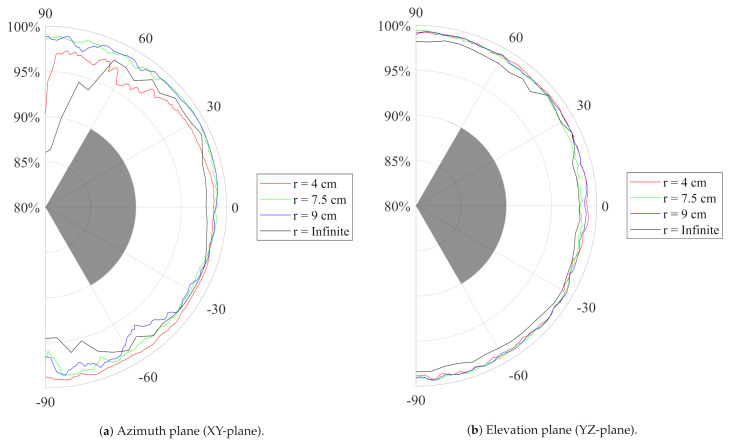
Measured System Fidelity Factor (SFF) of the standalone ntenna as a function of the respective angle. Different bending radii *r*, frequently encountered on the human body, are applied. As a reference, the SFF > 90% goal is marked in grey.

The receive antenna was rotated in its H-plane and E-plane, as shown in the top view on Figure 2, to record the transfer function of the entire system for every investigated position in a frequency band stretching from 1 GHz to 18 GHz with a step size of 50 MHz. The system impulse response was then found by Fourier transforming this transfer function. Next, as an input signal $T_{s}\left(t\right)$, the default output pulse of the DW1000 localization IC when operating in Channel 2 was selected. This input signal was then convoluted with the impulse response obtained from the aforementioned measurements, resulting in the pulse $R_{s}\left(t\right)$ as obtained at the port of the receive antenna. Finally, the SFF was obtained by taking the maximum of the normalized cross correlation of both signals, as described in [36]. Figure 6 shows the resulting SFF as a function of the respective rotation angle. To prove the mechanical flexibility, the antenna was subjected to mechanical bending along the x-axis in the H-plane and with different bending radii *r*, frequently encountered on the human body. It is clear that, for all directions within $60^{\circ }$ of the antenna’s broadside, the SFF stays well above 90%.

**Figure 7:**
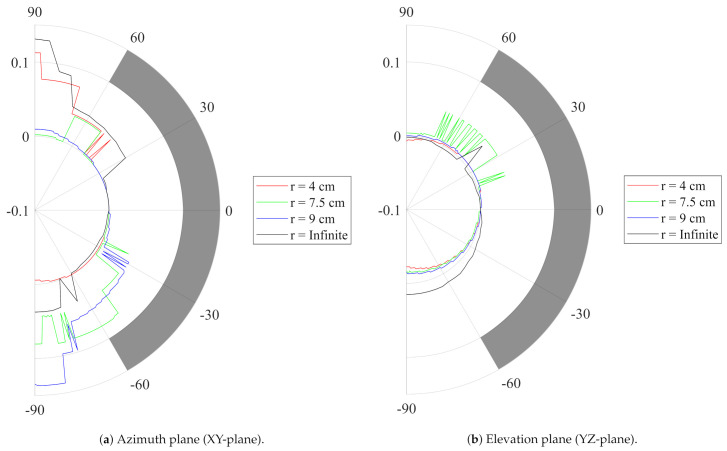
Measured Distance Estimation Error (DEE) (m) of the standalone antenna as a function of the respective angle. The broadside of the antenna radiation pattern is defined as having 0 cm deviation. Different bending radii *r*, frequently encountered on the human body, are applied. As a reference, the DEE < 10 cm goal is marked in grey

The time index at which the maximum of the normalized cross correlation of both signals is obtained depends on the relative orientation between the transmit and receive antennas. Therefore, as this time index defines the time of arrival of the signal pulse, the antenna orientation causes a distance estimation error. Figure 7 reveals that, in both investigated slices, for all directions within $60^{\circ }$ of the antenna’s broadside and for the same bending radii *r*, the maximum distance estimation error introduced by the antenna remains below the 5 cm predicted by simulations, which is smaller than the targeted 10 cm positioning error as generated by the DW1000 IC itself [13].

A comparison of UWB textile antennas is provided in Table 1. This table shows conventional antenna parameters as well as system-level parameters relevant to positioning, such as the pulse distortion, quantified by either the Fidelity Factor (FF) or the System Fidelity Factor (SFF) [37], dependent on the originally provided parameter. The proposed antenna has a lower impedance matching bandwidth in comparison to the other cited works, which are designed to cover the entire Federal Communications Commission (FCC) band. Nevertheless, it is sufficient to cover two IEEE802.15.4 UWB channels, while the antenna’s radiation efficiency is significantly better and exhibits a stable radiation pattern in proximity of the human body. Furthermore, the proposed antenna features excellent pulse distortion behavior.

### 3.2. System Performance

This section examines the measurements where the flexible PCB is integrated on the backplane of the adjusted textile antenna discussed in Section 2.3. Section 3.2.1 elaborates on the measured power pattern of the system and compares it to the radiation pattern of the standalone antenna. The results of the body-worn positioning experiment are shown in Section 3.2.2. Finally, the measured energy consumption of the system and the effect of power saving measures on battery life are discussed in Section 3.2.3.

#### 3.2.1. Radiated Power Pattern

**Figure 8:**
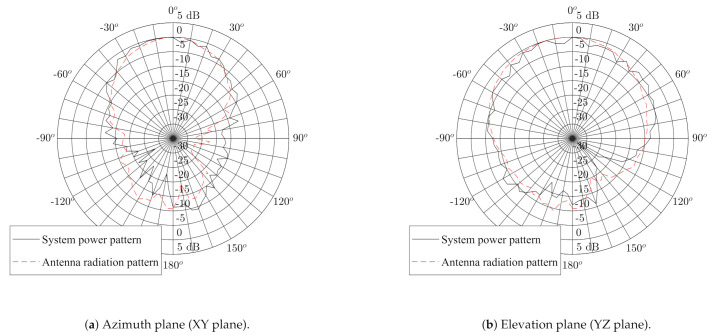
Received radiation pattern (black line) when the DW1000 is configured in continuous wave mode in Channel 2 overlayed with the measured radiation pattern published in [26] (dashed red line). For comparison, both patterns are normalized to 0 dB broadside gain.

The measurement results from [26] show that the standalone antenna possesses a single-lobe directional radiation pattern with a front-to-back ratio (FTBR) between 11.0 dB and 11.2 dB over the 3744 MHz to 4243.2 MHz band, which forms Channel 2 of the IEEE 802.15.4-2011 standard. We now verify whether the radiation pattern of the sensor system integrated on the backplane of the adjusted textile antenna possesses a similar FTBR, which is most desirable in WBAN systems, as motivated in Section 2.3. Therefore, the DW1000 IC was configured to transmit in continuous wave mode in Channel 2. The system is then placed on the rotor mount of an Orbit/FR DBDR antenna positioning system and measured using a R&S 1093.44995K07 FSV spectrum analyzer, resulting in the normalized power pattern shown in Figure 8. The power pattern is directive with a maximum along the ($-5^{\circ }$;$0^{\circ }$) azimuth-elevation direction, with a FTBR of 11.2 dB. The smoothed beam pattern’s 3 dB beamwidth measures $90^{\circ }$ in the elevation slice and $55^{\circ }$ in the azimuth slice. Figure 8 further exhibits a clear similarity between the normalized power pattern of the complete system, shown in Figure 4b, and the normalized radiation pattern of the standalone textile antenna in [26]. Combined with the limited influence of human body proximity on the radiation pattern of cavity-backed slot antennas [24], this similarity leads to the conclusion that the system is suitable for deployment on a human body. Therefore, the system should be able to reliably connect to any external localization infrastructure, which is further investigated in Section 3.2.2.

#### 3.2.2. Body-Worn Positioning Experiment

**Figure 9:**
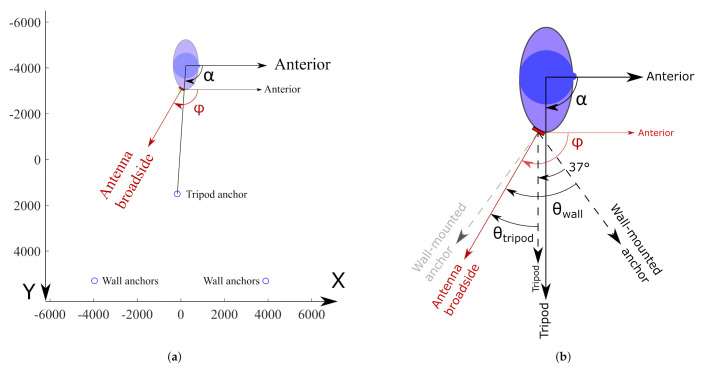
Body-worn positioning experiment. *α* is the angle between the user’s anterior direction and the direction of the tripod. *ϕ* is the angle between the user’s anterior direction and the textile antenna’s broadside orientation. (**a**) Anchor and test subject (not to scale) positions in mm with the anchor positions displayed as blue circles. Both anchors at the highest Y coordinate consists of two anchor nodes stacked on top of each other. For exact coordinates, please refer to Table 2. (**b**) Investigation of connectivity between the tripod mounted anchor and the sensor module. The sensor module was deployed at the body position characterized by *ϕ* = 120°. The tripod mounted anchor is located in the azimuthal direction for which *α* = 90°. The antenna’s broadside deflection with respect to the tripod mounted anchor is therefore *θ_tripod_* = +30°. The precise locations of the anchors are given in Table 2.

A body-worn localization system should be able to provide adequate quality-of-service, regardless of the user’s orientation with respect to the surrounding localization infrastructure. Inevitably, due to the body shadowing effect and given that the system employs a directional antenna optimized to counter this effect, not all anchor positions can be reached simultaneously by the same antenna. Therefore, multiple antenna positions on the wearer’s body should be used [42], as every direction around the user’s body must be serviced by at least one body-worn antenna element for all positions and orientations that the user might be in. To investigate this, a system consisting of multiple wearable sensor nodes was deployed at diverse locations around the waist of a male test person having a size of 1.90 m and a mass of 85 kg. This is described in Figure 9. Figure 9a shows the layout of the experimental setup. Note that both anchor nodes positioned on the bottom of the figure each contain two anchor nodes, located on top of each other, approximately 2.2 m apart. The precise locations of all anchor nodes, including the tripod mounted anchor, are constant throughout all experiments and have been determined using the MOCAP system [43]. They are described in Table 2. Figure 9b elaborates on the angles used to describe the positioning experiment. The angle between the user’s anterior direction and the direction of the tripod is given by α. The position of the textile antenna on the user’s body is characterized by the angle ϕ between the user’s anterior direction and the textile antenna’s broadside direction.

**Figure 10:**
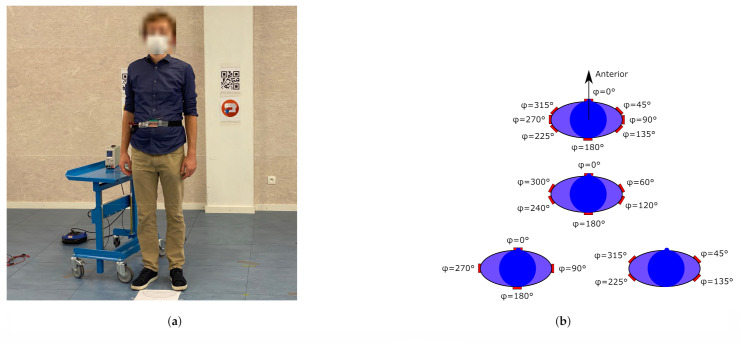
Deployment on the user’s body. (**a**) Test subject fitted with the belt hosting the sensor node. This is the on-body deployment position for $\phi =0^{\circ }$. The system is powered by an external power source for measurement purposes. (**b**) Some scenarios for 8 (top), 6 (middle), and 4 (bottom) body-deployment positions. Two possible configurations for 4 body-deployment positions are provided.

To facilitate testing, the system is attached to a belt, as shown in Figure 10a, allowing it to be shifted around the test person’s waist between the different body-deployment locations under study visualized in Figure 10b. At a distance of 4.53 m to the test person, an anchor node was mounted on a tripod. The sensor system was then implemented as a tag in the WiPos (Wireless Positioning) system [44]. It periodically transmited a packet every 67 ms using the configurations listed in Table 3.

Practical scenarios often involve anchor nodes that are deployed close to the ground or above its users’ heads. Table 2 shows that, in the WiPos test setup, anchor nodes were deployed at such locations. If the vertical plane beamwidth of the textile antenna of the body-worn wireless sensor node are too narrow, approaching these anchor nodes could induce connectivity loss since it would set up a wireless link with the anchor nodes along a direction where the textile antenna’s gain is low. Therefore, the antenna is deployed such that its E-plane, with a smoothed beamwidth of $90^{\circ }$, coincides with the vertical plane, since Figure 8 shows that the antenna’s E-plane beamwidth is larger than its H-plane beamwidth, which equals $55^{\circ }$. The measurements reveal that the minimum horizontal distance between the sensor system and the aforementioned anchor nodes in which a connection can be maintained equals zero.

Furthermore, in a practical application, every wireless sensor node’s position is responsible for covering an azimuthal section of the wearer’s surroundings. The nodes’ deployment positions should be chosen such that these sections provide connectivity with external hardware over the full $360^{\circ }$ of azimuthal coverage around the wearer. To verify this for any chosen deployment position, the connectivity should be investigated not only for the case in which the antenna’s broadside is directed towards the tripod-mounted anchor but also for when the broadside points away from the tripod’s direction at an azimuthal deflection angle $\theta_{tripod}$, as displayed in Figure 9b. In this experiment, a value $\theta_{tripod}=30^{\circ }$ in both directions is investigated. When the sensor system is placed at a position for which $\phi =0^{\circ }$, as illustrated in Figure 10b, the maximum value for θ at which connectivity is maintained is found to be slightly larger than $60^{\circ }$.

To quantify the number of body-deployment positions necessary to provide connectivity in all directions around the user, the fraction of packets received by the tripod mounted anchor with respect to the packets sent by the sensor node was investigated. Therefore, the wireless sensor node was deployed at the different body positions shown in Figure 10b. Table 4 lists the percentage of packets received by the tripod mounted anchor when the textile antenna’s broadside was directed towards the tripod ($\theta_{tripod}=0^{\circ }$) or was azimuthally deflected $30^{\circ }$ away from it ($\theta_{tripod}=\pm 30^{\circ }$). For every measurement, the test subject was moved to ensure that the wireless sensor node was located at the coordinates specified in Table 2. As Table 4 shows, the user’s arms shadow the sensor node for the body positions associated with $\phi =120^{\circ }$ and $\phi =240^{\circ }$, although connectivity data for the positions associated with $\phi =135^{\circ }$ and $\phi =225^{\circ }$ strongly suggests the viability of solutions with only 6 deployment positions. However, using 8 body-deployment positions distributed evenly around the test person’s waist is sufficient to provide coverage in all directions. Indeed, Table 4 shows that all deployment positions with a broadside direction in which the sensor system fails to connect with the tripod mounted anchor are backed up by a successful connection from the neighboring deployment position. For example, an anchor node positioned such that $\alpha =120^{\circ }$ is not serviced by a sensor node placed at the body position associated with $\phi =90^{\circ }$, since in this position, Table 4 reveals that, for $\theta_{tripod}=-30^{\circ }$, no packets have been received. However, the neighboring body-deployment position, where $\phi =135^{\circ }$, provides coverage over all investigated θ angles, which include the direction for which $\alpha =120^{\circ }$. Therefore, it covers this direction, which was shadowed for the $\phi =90^{\circ }$ body-deployment position.

When considering solutions with fewer than 6 body-deployment locations, the azimuthal sections that need to be covered by each body-deployment position become wider than the $60^{\circ }$ coverage, represented by the data provided in Table 4. However, Table 2 and Figure 9 reveal the presence of four other anchor nodes in the Y direction at an azimuth angle of $\pm 37^{\circ }$ from the tripod direction. Therefore, the azimuthal difference between the antenna’s broadside direction and the direction of these wall-mounted anchor nodes visualized in Figure 9b totals $\theta_{wall}=\theta_{tripod}+37^{\circ }$. This provides extra connectivity data, presented in Table 5. Here, for all investigated body-deployment locations, the fraction of the amount packets received on the two wall-mounted anchor nodes located at an azimuthal deflection $\theta_{wall}=67^{\circ }$ with respect to the textile antenna’s broadside direction, is displayed. From Table 5, no suitable combination of fewer than 6 body-deployment locations can be found that provide coverage for all directions around the user. To qualify a body-deployment position for a certain ϕ as suitable, the connectivity between the anchor nodes and the sensor system should be reliable for all investigated elevation angles.

#### 3.2.3. Power Use

Since battery capacity takes up a lot of the available weight and space budget in a body-worn scenario, efficient power use is of great importance. Given that the DW1000 localization IC also acts as a transceiver, this component will be the most important consumer of power. Considering that the DC/DC converter exhibits an efficiency higher than 90% under the operating conditions [45] and that the other components’ power consumptions are dwarfed by that of the DW1000’s transceiver [27,28,29], it is primarily the duty cycle at which the DW1000’s transceiver is active, for example, by sniffing for packets, which determines the system battery’s lifespan. It is, therefore, necessary that a practical system would employ a timeslot-based communication schedule, as is required by the IEEE 802.15.4-2011 standard.

To provide a few measured indicative values for autonomy of the system, the system was configured in some representative scenarios, which are summarized in Table 6. In all scenarios, the DW1000 localization IC is configured according to the settings listed in Table 3. The device operates in Channel 2 of the IEEE 802.15.4-2011 standard. In the first scenario from Table 6, the system was configured in its most power-hungry state. Intentionally, none of the power-conserving mechanisms and methods suggested by the manufacturer were applied, which results in the microcontroller returning the localization IC to the receive mode immediately after it completed a packet reception. In this worst-case setting, the system spends 700 mW of power. Currently, available flexible batteries [46] with a capacity of 200 mAh and a footprint comparable to this system would make the system last for only 1.42 h. Clearly, a degree of power conservation should be applied for practical application of the system. The second scenario from Table 6 is the standby situation: all peripheral components are powered up but idle. The microcontroller internally performs operations. Table 6’s third scenario constitutes a basic use of the localization IC, where it periodically transmits dummy payloads of 17 bytes long at an interval of 16 packets per second. The table shows that the difference in the system’s measured average current is below 1 mA, as measured on the power supply. On a 200 mAh battery, this provides to the system an autonomy of 13.3 h, even without the use of any component’s sleep mode. To further limit power use, UWB Medium Access Control (MAC) protocols such as [47] can be applied, which further save power by periodically disabling the UWB radio. As such, 13.3 h is still a pessimistic value, leading to the conclusion that the system’s power use is suitable for a WBAN system.

## 4. Conclusions

A complete and mechanically flexible wireless body area network sensor system was proposed. The system consists of a full-textile cavity-backed slot antenna designed for use in Channels 2 and 3 of the IEEE 802.15.4-2011 standard. By measuring the impulse response of the measurement setup containing two standalone antennas, the system fidelity factor was proven to be well above the 90% criterion commonly used in localization. Furthermore, the distance estimation error, resulting from the angle-dependent phase difference between a wave incoming from broadside with respect to a wave incoming from a given incident angle, proves to be below 5 cm in both the azimuth plane and the elevation plane of the antenna. This is lower than the error introduced by the localization IC. The antenna was modified slightly to enable on its feed plane the integration of a mechanically flexible PCB, which hosts a temperature, relative humidity, and air pressure sensor; a DW1000 Impulse-Radio Ultra-Wideband localization IC; and a 4 MB flash memory. Measurements in which the transceiver was configured in continuous wave mode prove that the presence of the PCB on the feed plane of the antenna has little influence on the antenna’s radiation pattern. A directive power pattern with a front-to-back ratio of 11.2 dB was obtained. Its shape is very similar to the radiation pattern of the standalone textile antenna. The antenna’s directive radiation pattern requires it to be deployed at multiple deployment locations on the wearer’s body. By relying on eight deployment positions, coverage in all directions is provided with ample redundancy. The measurements suggest that six deployment positions suffice as well if these positions are chosen carefully, to avoid shadowing by the wearer’s arms. Configurations with fewer deployment positions were proven not to be feasible. Analysis of the localization accuracy of a system employing multiple collaborating nodes, along with a formal investigation of optimal body-deployment positions are outside the scope of this paper. They will be considered in future research. The power use of the complete sensor system heavily depends on the duty cycle at which the transceiver is activated. In a worst-case testing scenario in which 16 packets per second were sent, the measurements indicate that a system autonomy of 13.3 h can be obtained from a 5 V, 200 mAh flexible battery. The innovations in this paper include the co-design of a low-power, comprehensive mechanically flexible PCB featuring both digital and time-domain-sensitive analog parts with a mechanically flexible textile antenna for UWB localization. Furthermore, the suitability of full-textile cavity-backed slot antennas for localization purposes was demonstrated by measurement of both the antenna’s pulse distortion and phase center shift. This system acts as a proof of concept for the implementation of electronics for localization in a complete and fully flexible sensor system in textile.

## Figures and Tables

**Table 1:** Comparison with other ultra-wideband (UWB) antennas throughout literature.

Ref.	Antenna Topology	Maximum Gain	Impedance Match [GHz]	Fidelity in Free Space	Approx. −3 dB Beamwidth	Antenna Efficiency
[38]	Arc-shaped patch	6 dBi	3.8–6, 6.6–8.3, 8.6–9.8	0.78–0.92 (SFF)	30${}^{\circ }$–60${}^{\circ }$	20–40%
[39]	Octagonal monopole	1.1–7.2 dBi	3.2 –>11	>96% (SFF)	<20${}^{\circ }$–70${}^{\circ }$	not evaluated
[40]	Annular slot	not evaluated	3.2–9.9	80–100% (FF)	60${}^{\circ }$–140${}^{\circ }$	not evaluated
[41]	Splined monopole	5 dBi	3 –>16	not evaluated	80${}^{\circ }$– omnidirectional	5–40%
This antenna	Cavity- backed slot antenna	>6.4 dBi	3.7–4.8	>97% (SFF)	75${}^{\circ }$–95${}^{\circ }$	78–91%

**Table 2:** The positions of the anchor nodes shown in Figure 9a with respect to the measurement space center.

Device	x (mm)	y (mm)	z (mm)
Anchor Wall Bottom Right	−3980	5302	440
Anchor Wall Bottom Left	3886	5318	439
Anchor Wall Top Right	−3980	5302	2647
Anchor Wall Top Left	3886	5318	2653
Anchor Tripod Mounted	−164.6	1504.6	1205.4
Sensor system, body-worn	−100	−3000	1200

**Table 3:** Settings used in the on-body positioning experiment.

Parameter	Setting
UWB Channel	2
Bitrate	850 kbps
Pulse Repetition Frequency (PRF)	64 MHz
Preamble length	1024
Preamble code	10

**Table 4:** Percentage of packets received by the anchor on the fixed tripod mount for different body-deployment positions characterized by ϕ and for different deflections $\theta_{tripod}$ between the textile antenna’s broadside orientation with respect to the direction of the tripod mount. As visualized in Figure 9b, the test person was oriented such that $\alpha =\phi -\theta_{tripod}$.

		6 Body Positions					
		**Percentage of Packets Received**			**Number of Sent Packets**		
	$\theta_{tripod}$	−30${}^{\circ }$	0${}^{\circ }$	30${}^{\circ }$	−30${}^{\circ }$	0${}^{\circ }$	30${}^{\circ }$
ϕ							
0${}^{\circ }$		100.00%	100.00%	100.00%	184	334	152
60${}^{\circ }$		100.00%	100.00%	100.00%	112	200	149
120${}^{\circ }$		62.89%	0.00%	0.00%	97	69	101
180${}^{\circ }$		100.00%	100.00%	100.00%	114	115	175
240${}^{\circ }$		0.00%	100.00%	100.00%	141	123	123
300${}^{\circ }$		100.00%	100.00%	100.00%	132	157	129
		**8 Body positions**					
		**Percentage of Packets Received**			**Number of Sent Packets**		
	$\theta_{tripod}$	−30${}^{\circ }$	0${}^{\circ }$	30${}^{\circ }$	−30${}^{\circ }$	0${}^{\circ }$	30${}^{\circ }$
ϕ							
0${}^{\circ }$		100.00%	100.00%	100.00%	184	334	152
45${}^{\circ }$		100.00%	100.00%	100.00%	148	189	140
90${}^{\circ }$		0.00%	100.00%	100.00%	117	150	145
135${}^{\circ }$		100.00%	100.00%	100.00%	81	79	116
180${}^{\circ }$		100.00%	100.00%	100.00%	114	115	175
225${}^{\circ }$		100.00%	100.00%	100.00%	131	140	90
270${}^{\circ }$		100.00%	100.00%	99.24%	161	108	131
315${}^{\circ }$		100.00%	100.00%	100.00%	171	164	164

**Table 5:** Percentage of transmitted packets received by the two wall-mounted anchor nodes located in the opposite direction of the textile antenna’s broadside rotation for different body-deployment positions. With respect to the user’s anterior direction, the connectivity directions α represented by these wall anchors are given by $\alpha =\phi -\theta_{wall}$, as can be seen on Figure 9b. The table shows that no combination of fewer than 6 body-deployment locations ϕ can be found in which the node can make a reliable connection with the anchors deployed in all investigated elevation angles.

		Percentage of Packets Received			
$\theta_{\mathrm{wall}}$		$-67^{\circ }$		$67^{\circ }$	
	Receiving Anchor	Top Right	Bottom Right	Top Left	Bottom Left
ϕ					
0${}^{\circ }$		1.11%	95.58%	0.00%	0.00%
45${}^{\circ }$		21.92%	97.26%	0.00%	12.50%
60${}^{\circ }$		0.00%	17.27%	0.00%	0.00%
90${}^{\circ }$		0.00%	0.00%	80.28%	100.00%
120${}^{\circ }$		3.19%	56.38%	0.00%	0.00%
135${}^{\circ }$		90.91%	98.70%	0.00%	0.00%
180${}^{\circ }$		0.00%	0.90%	0.00%	85.55%
225${}^{\circ }$		0.00%	0.00%	0.00%	96.51%
240${}^{\circ }$		0.00%	0.00%	95.83%	100.00%
270${}^{\circ }$		0.00%	30.82%	0.00%	0.00%
300${}^{\circ }$		78.46%	99.23%	0.00%	0.00%
315${}^{\circ }$		36.53%	97.62%	0.00%	32.92%

**Table 6:** System power use in various scenarios and associated lifetime on a 5 V, 200 mAh battery.

Scenario	Measured Average Current	Battery Power Use	Battery Lifetime
Continuous RX	140 mA	700 mW	1.42 h
Microcontroller active, DW1000 idle	15 mA	75 mW	13.3 h
Microcontroller active, DW1000 transmitting @ 16 packets/s	15 mA	75 mW	13.3 h

## Data Availability

Not applicable.
